# A Knockout of the *Tsg101* Gene Leads to Decreased Expression of ErbB Receptor Tyrosine Kinases and Induction of Autophagy Prior to Cell Death

**DOI:** 10.1371/journal.pone.0034308

**Published:** 2012-03-30

**Authors:** Chantey R. Morris, Marissa J. Stanton, Karoline C. Manthey, Keon Bong Oh, Kay-Uwe Wagner

**Affiliations:** 1 Eppley Institute for Research in Cancer and Allied Diseases, University of Nebraska Medical Center, Omaha, Nebraska, United States of America; 2 Department of Pathology and Microbiology, University of Nebraska Medical Center, Omaha, Nebraska, United States of America; Ohio State University, United States of America

## Abstract

The *Tumor Susceptibility Gene 101* (*Tsg101*) encodes a multi-domain protein that mediates a variety of molecular and biological processes including the trafficking and lysosomal degradation of cell surface receptors. Conventional and conditional knockout models have demonstrated an essential requirement of this gene for cell cycle progression and cell viability, but the consequences of a complete ablation of Tsg101 on intracellular processes have not been examined to date. In this study, we employed mouse embryonic fibroblasts that carry two *Tsg101* conditional knockout alleles to investigate the expression of ErbB receptor tyrosine kinases as well as stress-induced intracellular processes that are known to be associated with a defect in growth and cell survival. The conditional deletion of the *Tsg101* gene in this well-controlled experimental model resulted in a significant reduction in the steady-state levels of the EGFR and ErbB2 but a stress-induced elevation in the phosphorylation of mitogen activated protein (MAP) kinases independent of growth factor stimulation. As part of an integrated stress response, Tsg101-deficient cells exhibited extensive remodeling of actin filaments and greatly enlarged lysosomes that were enriched with the autophagy-related protein LC3. The increase in the transcriptional activation and expression of LC3 and its association with Lamp1-positive lysosomes in a PI3K-dependent manner suggest that Tsg101 knockout cells utilize autophagy as a survival mechanism prior to their ultimate death. Collectively, this study shows that a knockout of the *Tsg101* gene causes complex intracellular changes associated with stress response and cell death. These multifaceted alterations need to be recognized as they have an impact on defining particular functions for Tsg101 in processes such as signal transduction and lysosomal/endosomal trafficking.

## Introduction

The *Tumor Susceptibility Gene 101* (*Tsg101*) was originally identified in transformed NIH3T3 cells using a random antisense knockdown approach [1]. This gene encodes a multi-domain protein that mediates a variety of molecular and biological processes. The earliest examination of structural features and cellular functions of Tsg101 showed that this protein may play a role in the regulation of transcription [2], [3] as well as ubiquitination [4], [5]. Following the cloning and sequencing of the entire *Tsg101* gene and revising its genomic architecture and coding sequence [6], [7], we performed a computational analysis to identify homologous proteins using the Sequence Alignment and Modeling System (SAM T98) [8]. While this preliminary study confirmed that the N-terminal portion of Tsg101 is similar to ubiquitin conjugating enzymes, we also found that the predicted Tsg101 structure exhibited an even stronger homology to dynactin, which is involved in the movement of vesicles and organelles along microtubules (McQueen and Wagner, unpublished). Since its classification as the mammalian ortholog of the yeast Vps23p/Stp22p by Li et al. [9] and Babst et al. [10], Tsg101 has been recognized as an integral component of the endosomal sorting complex required for transport (ESCRT)-I, which is involved in the ubiquitin-dependent sorting of proteins into endosomes.

The phenotypic examination of the first conventional knockout model by Ruland and colleagues [11] showed that Tsg101 is essential for early embryonic development. To investigate the role of this gene during normal organogenesis and carcinogenesis, our laboratory generated Tsg101 conditional knockout mice that allow a temporally and spatially controlled ablation of this gene in postnatal animals and derived cell types. In sharp contrast to the previously proposed tumor suppressive role of this gene, mice that lack *Tsg101* in various tissues including the mammary epithelium did not develop preneoplastic lesions and cancer [12], [13]. Moreover, the Cre/loxP-based conditional deletion of the promoter and first coding exon of the *Tsg101* gene resulted in a p53-mediated cell cycle arrest. Subsequently, Tsg101-deficient cells undergo apoptosis that is independent of functional p53, p19^Arf^, or p21^Cip/Waf^ [12], [14]. The ubiquitously expressed *Tsg101* gene possesses all the hallmarks of a housekeeping gene [6], and the collective studies on Tsg101 knockout cells confirmed that the essential functions of this gene for cell proliferation and survival seem to apply to all cell types that have been examined thus far including those that have undergone neoplastic transformation [14]–[16]. In support of this notion, recent work from various research teams including our own shows that Tsg101 is overexpressed rather than lost in a subset of breast, lung, thyroid, ovarian, and colon cancers [17]–[21].

As a member of the heterotetrameric ESCRT-I complex, Tsg101 associates with other vacuolar sorting proteins [Vps28, Vps37, and the multivesicular body protein 12 (MVB-12)] and facilitates the binding and sorting of ubiquitinated cargo proteins through its ubiquitin-conjugating enzyme E2 variant (UEV) domain. Here, Tsg101 is suggested to mediate multifaceted intracellular functions such as the downregulation of ubiquitinated cell surface receptors, in particular EGFR [10], [22]–[24], autophagic clearance of certain proteins aggregates [25], cytokinesis [26], and viral egress from infected cells [27], [28]. Despite a wealth of information about these proposed intracellular functions of the Tsg101 protein, it is still a conundrum why a knockout of the mammalian *Tsg101* gene causes cell cycle arrest and cell death instead of accelerated growth and tumor formation.

The aim of this study was to examine the effect of a conditional deletion of the *Tsg101* gene on stress-induced intracellular processes that might be associated with a defect in growth and cell survival as well as the expression of ErbB receptor tyrosine kinases, which have never been examined in Tsg101 knockout cells. Collectively, the finding show that the deletion of Tsg101 resulted in a significant reduction in the steady-state levels of the EGFR and ErbB2. In addition, Tsg101 knockout cells developed enlarged lysosomes that were enriched with the autophagy-related protein LC3. A significant increase in the expression of LC3 and its association with Lamp1-positive lysosomes in a PI3K-dependent manner suggest that cells lacking Tsg101 seem to utilize autophagy as a survival mechanism prior to their ultimate death.

## Results

### Tsg101 deficiency leads to a decline in EGFR expression and signaling but induces a growth-factor independent phosphorylation of Erk1/2

**Figure 1:**
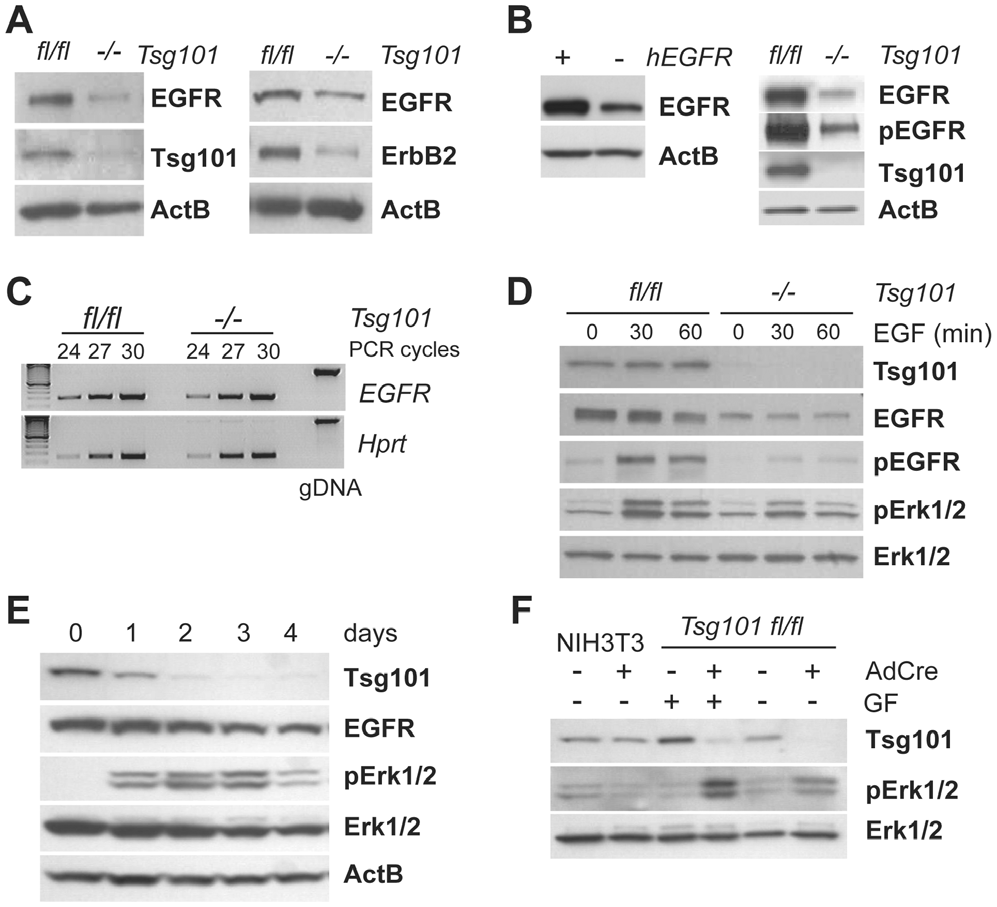
A conditional knockout of Tsg101 results in reduced expression of EGFR and ErbB2 but a stress-induced activation of Erk1/2 in a growth factor independent manner. **A**., **B**. Western blot analyses to assess the steady-state levels of endogenous EGFR and ErbB2 (A) as well as exogenous human EGFR in Tsg101 (B) conditional knockout fibroblasts (−/−) and their isogenic controls expressing Tsg101 (fl/fl). **C**. Semi-quantitative RT-PCR to examine the transcriptional activation of the endogenous *EGFR* gene in response to Tsg101 ablation. Genomic DNA (gDNA) was used as a control for the PCR with exon-specific primers that span intronic sequences of the mouse *EGFR*. **D**. EGFR and Erk1/2 expression and activation in response to EGF stimulation (0, 30, and 60 min) in cells lacking Tsg101 and their isogenic controls. **E**. Western blot analysis to assess immediate and long-term changes in EGFR expression and activation of Erk1/2 between one and four days following the conditional deletion of *Tsg101*. **F**. Analysis of Erk1/2 phosphorylation in the presence and absence of growth factors (GF) in response to Tsg101 deficiency.

We previously generated immortal mouse embryonic fibroblast (MEFs) cell lines that carry two conditional knockout alleles of the *Tsg101* gene (*Tsg101^fl/fl^*) that can be excised from the genome upon expression of Cre recombinase [12], [14]. Infection of these cells with a pBabe-Cre retroviral vector and selection with puromycin led to a substantial downregulation of the Tsg101 protein (Fig. 1). Since Tsg101 has been suggested to regulate the expression and trafficking of active ErbB receptor tyrosine kinases [23], [24], we first examined the levels of endogenous EGFR and ErbB2. In contrast to these previous reports, we observed a consistent and substantial decline in the steady-state levels of these receptors in Tsg101 knockout cells compared to control MEFs (Fig. 1A; also see 1D and 1E). A reduction in the total and phosphorylated levels of the EGFR was also detected in Tsg101 knockout cells that stably express exogenous human EGFR (Fig. 1B). The reduction in the steady state-levels of EGFR were likely caused by postranslational processes since Tsg101 deficiency did not affect the level of receptor mRNA as determined by a semi-quantitative RT-PCR assay (Fig. 1C). This was also the case for the exogenous human *EGFR* message, which was under control of a constitutively-active CMV promoter (data not shown).

To assess whether the ligand-induced degradation of EGFR is affected by the lack of Tsg101, we treated control and Tsg101 knockout cells with EGF for 30 and 60 minutes and examined the expression of the EGFR (total and phosphorylated) by western blot analysis. The addition of the ligand led to the expected decline in the level of EGFR in wildtype control cells, and a slight reduction in EGFR expression from a lower baseline was also seen in cells that lack Tsg101 (Fig. 1D). Concomitant with a decline in the steady-state levels of the receptor, we observed a decrease in EGF-induced activation of Erk1/2 in Tsg101-deficient cells compared to their isogenic controls that express endogenous Tsg101. In conclusion, the reduction in the steady-state levels of EGFR in Tsg101 knockout cells was not caused by an enhanced ligand-induced degradation of the receptor.

Previous studies have reported that an siRNA-mediated knockdown of Tsg101 led to elevated levels of EGFR due to enhanced recycling or retention of the receptor within early endosomes [23], [24]. To further examine a cause for our unexpected findings, we asked whether the reduced expression of the EGFR in the Tsg101 null cells might be due to differences in the experimental design, i.e. 72 hrs after siRNA treatment in previous reports versus 72–96 hrs following expression of Cre and selection with puromycin in the conditional knockout model. To address this issue and assess the effects of an acute downregulation of Tsg101 without the use of a selection agent, we infected Tsg101 conditional knockout cells with an adenovirus expressing Cre recombinase (AdCre) and examined the steady-state level of the EGFR over a time course of four days. The expression of Tsg101 started to decline at day 1 following infection with AdCre and was nearly complete by day 3 (Fig. 1E). Loss of Tsg101 was accompanied by a gradual reduction in the expression of the EGFR with a slight delay, becoming apparent after day 2 and with a steady decline thereafter. The results of this experiment indicate that the reduction in EGFR is proportional to the gradual decrease in the level of Tsg101. It might be possible that the absence of a similar decline in EGFR expression in previous siRNA knockdown studies might be due to insufficient depletion of Tsg101. Interestingly, while the overall levels of Erk1/2 remained unchanged initially and declined at later time points upon deletion of Tsg101, we observed an increase in the level of phospho-Erk1/2 that peaked between days 2 and 3 followed by a decline at day 4 (Fig. 1E). Infection of NIH3T3 cells (wildtype for Tsg101) with AdCre did not lead to an elevation of activated MAP kinases (Fig. 1F, lanes 1 and 2), suggesting that the increase in pErk1/2 levels in AdCre-infected *Tsg101^fl/fl^* MEFs was not due to adenovirus infection per se but rather due to the lack of Tsg101.

The increase in the level of active Erk1/2 was observed at a time when EGFR levels were actually declining, and we therefore asked whether the elevation in the levels of pErk1/2 in Tsg101 knockout cells was dependent on the presence of growth factors in the medium. To address this issue, we examined the levels of active Erk1/2 in mock-infected compared to AdCre-infected cells that were maintained in the presence or absence of growth factors (Fig. 1F, lanes 3 through 6). While a higher level of pErk1/2 was seen upon deletion of Tsg101 in cells maintained in growth factor-containing media, a substantial increase in pErk1/2 levels was also detected upon Tsg101 deletion in growth factor-deprived cells. In conclusion, these findings suggest that loss of Tsg101 triggers the activation of ERKs, but this increase is not primarily the result of a sustained growth factor receptor signaling as reported earlier.

### A knockout of Tsg101 causes a remodeling of actin filaments as well as an enlargement of lysosomes without affecting the delivery Cathepsin D into these distended vesicles

**Figure 2:**
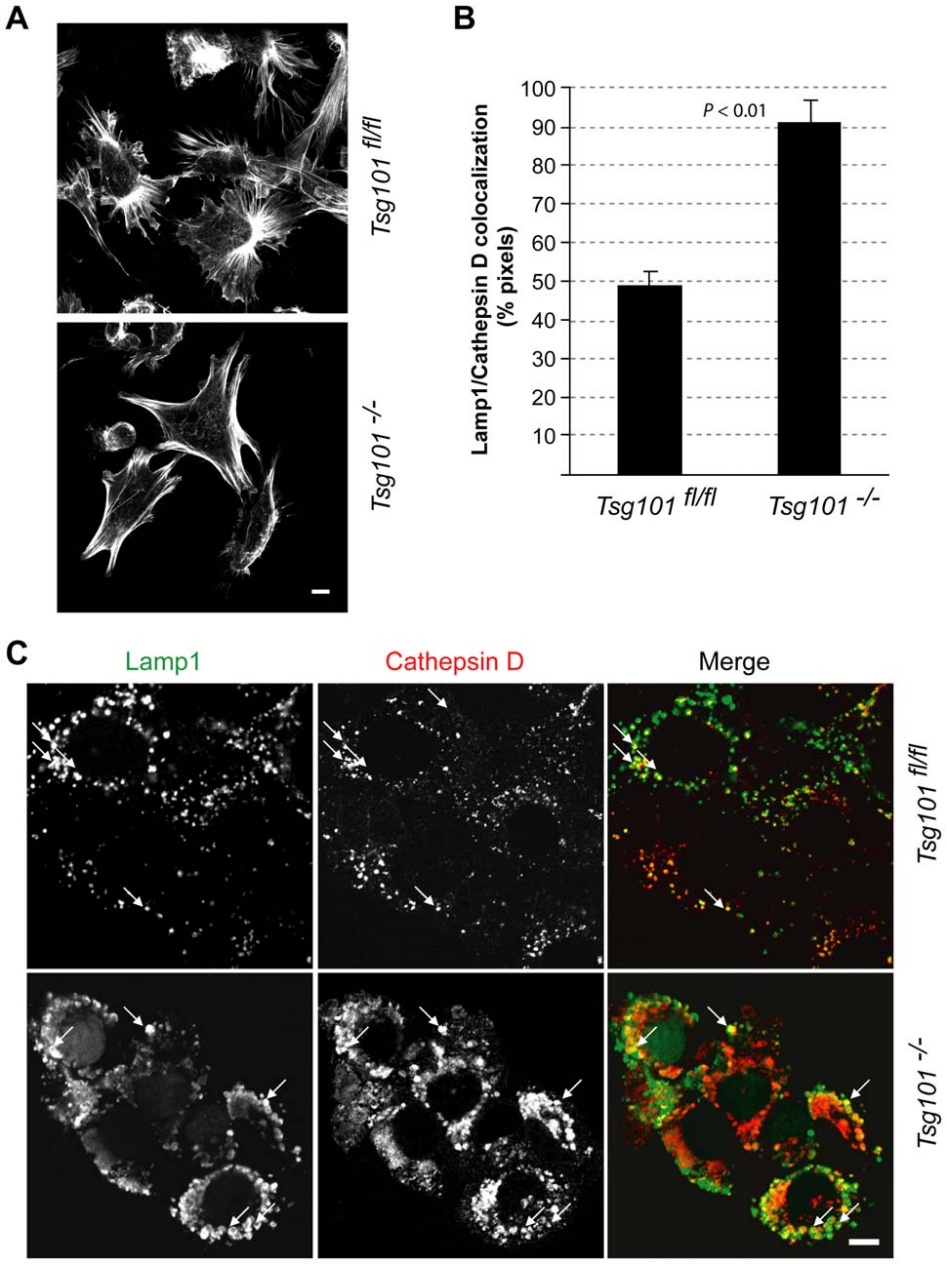
Remodeling of actin filaments and enlargement of lysosomes in response to the conditional deletion of the *Tsg101* gene. **A**. Confocal microscopic images of a phalloidin-based fluorescent staining of F-actin in Tsg101 conditional knockout cells (*Tsg101^−/−^*) and their controls (*Tsg101^fl/fl^*). **B**., **C**. Co-staining of Tsg101-deficient cells and their wildtype controls with antibodies against Lamp1 and Cathepsin D; panel B illustrates the quantitative analysis of the colocalization of both signals (*P* value, *t* test). Note that Cathepsin D, which was more abundant in Tsg101-deficient cells, was clearly present within the lumen of the enlarged lysosomes (panel C, arrows). Bar in panel C represents 10 µm.

The activation of the mitogen-activated protein (MAP) kinases in a growth-factor independent manner as well as the initiation of a cell cycle arrest are both indicative for a stress adaptation of cells following the deletion of *Tsg101*. The redistribution of actin filaments is another characteristic of stressed cells, and we therefore anticipated obvious changes in the architecture of the cytoskeleton in Tsg101 knockout cells. The phalloidin-based fluorescent staining of F-actin in Tsg101-deficient cells compared to wildtype controls (Fig. 2A) clearly shows that deficiency in Tsg101 led to a pronounced widespread reorganization of actin filaments with few branches. It has been proposed recently that F-actin remodeling is a necessary event that precedes the fusion of the autophagosome with the lysosome [29], which is a key event for the integrated stress response. Indeed, Tsg101-deficient cells exhibited greatly enlarged Lamp1-positive lysosomes that often had a ring-shaped appearance (Fig. 2C). Although it had been reported that a knockdown of Tsg101 caused multiple trafficking defects [23], [24], we observed that the routing of biosynthetic cargo from the Golgi and delivery of nascent lytic hydrolases to the lysosome were not impaired in cells that are conditionally deficient in Tsg101 (Fig. 2B, 2C). Cathepsin D, which appeared to be more abundant in Tsg101-deficient cells, was clearly present within the lumen of the enlarged lysosomes. In addition, lack of Tsg101 did not appear to affect the size or morphology of the cis-medial golgi as determined by staining with antibodies against giantin and GM130 (data not shown). Collectively, these observations suggest that a knockout of Tsg101 does not generally block the delivery of cargo to the lysosome, but it initiates a number of events including the remodeling of actin filaments and the enlargement of lysosomes that might be a consequence of an induction of autophagy.

### Elevated expression of the LC3 protein and its co-localization with lysosomes is indicative of the induction of macroautophagy in response to Tsg101 deficiency

**Figure 3:**
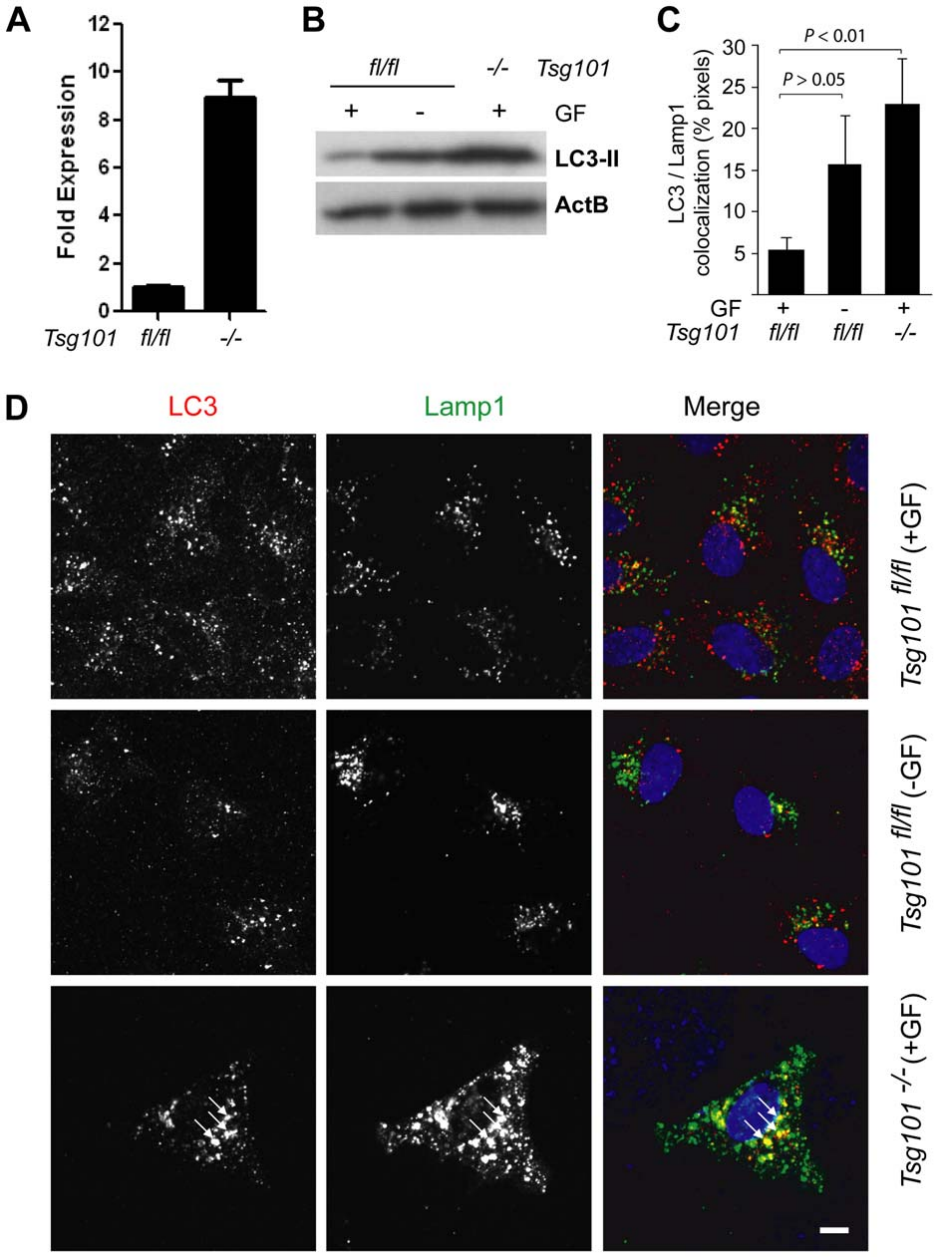
Tsg101 deficiency leads to induction of macroautophagy. **A**. Quantitative real-time RT-PCR to assess the transcriptional activation of the *Microtubule-associated protein 1/light chain 3* (*Map1lc3*) gene in Tsg101 conditional knockout cells and their wildtype controls. **B**. Western blot analysis to verify the increase in expression of LC3 in response to Tsg101 deficiency. Wildtype cells maintained in the absence of growth factors (GF) were used as a positive control. **C**., **D**. Analysis of confocal images of Tsg101 conditional knockout cells and their wildtype controls that were maintained in the presence or absence of growth factors (GF) prior to fixation and co-staining with antibodies against endogenous LC3 and Lamp1. Panel C illustrates the quantitative analysis of the colocalization of LC3 and Lamp1 (*P* value, *t* test). The bar in panel D represents 10 µm and arrows indicate areas of intensive co-localization of LC3 and Lamp1.

To assess whether Tsg101 deficiency leads to the initiation of macroautophagy, we examined the mRNA level of the *Microtubule-associated protein 1/light chain 3* (*Map1lc3*) gene as well as the expression and intracellular localization of its encoded protein, LC3. As a mammalian homolog of the yeast Atg8, LC3 is a constituent of the autophagosome. The quantitative RT-PCR showed that the transcriptional activation of the *Map1lc3* gene was approximately 8 to 10 times higher in Tsg101 knockout cells compared to their wildtype controls (Fig. 3A). Unlike in wildtype cells that were grown in the presence of serum, the withdrawal of growth factors led to the expected increase in the level of the LC3 protein (Fig. 3B). Similar to serum starvation, the conditional deletion of the *Tsg101* gene led to a significant elevation in LC3 expression despite the presence of growth factors.

**Figure 4:**
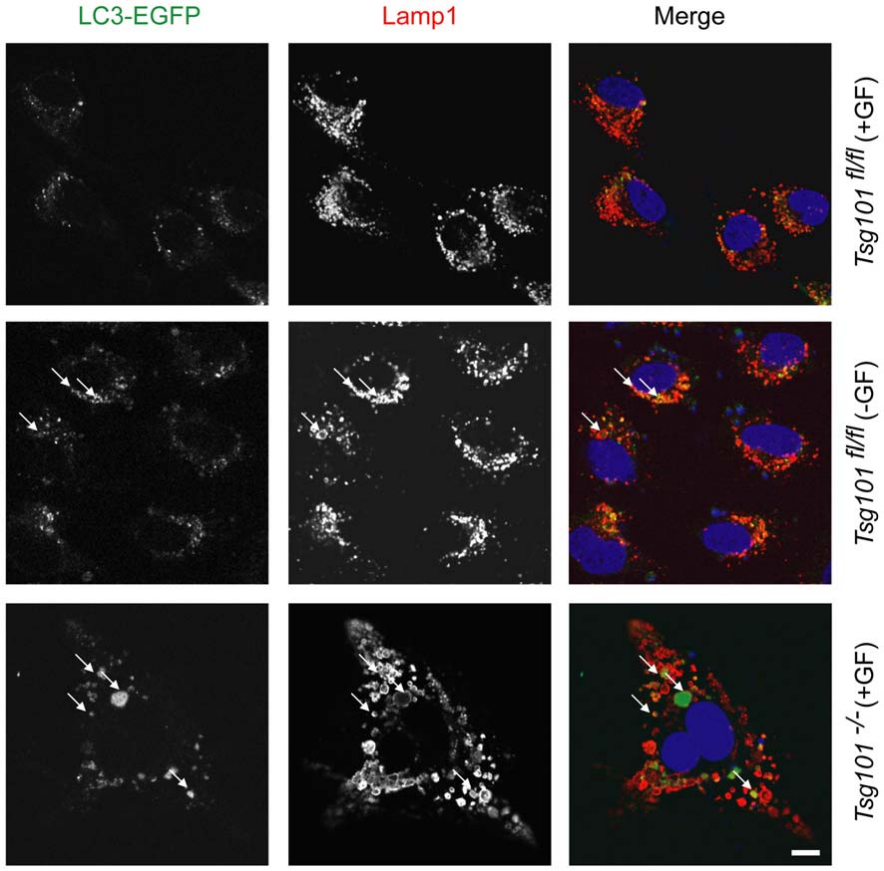
Accumulation of exogenous LC3-EGFP within the distended lumen of Tsg101 knockout cells. Confocal images of Tsg101 conditional knockout cells and their wildtype controls that were transfected with an LC3-GFP expression vector and maintained in the presence or absence of growth factors (GF). Cells were fixed and stained with an antibody against Lamp1; bar represents 10 µm.

Next, we used immunofluorescence staining and confocal microscopy to assess the intracellular distribution of endogenous LC3. A co-staining of this autophagy-related protein with a lysosomal marker clearly shows that LC3 is abundant and highly enriched within Lamp1-positive structures in cells that lack Tsg101 (Fig. 3C, 3D). To confirm these findings, we transfected Tsg101 knockout cells and their controls with a vector that expresses an EGFP-tagged LC3 protein. As anticipated, a moderate increase in the co-localization between LC3-EGFP and lysosomes was observed in wildtype cells that were maintained without growth factors (Fig. 4). The extent of the association between these markers was more pronounced in Tsg101 knockout cells due to the presence of greatly enlarged Lamp1-positive vesicles, which frequently exhibited an accumulation of LC3-EGFP within their lumen (Fig. 4, bottom panel, arrows). Collectively, the results of this study show that deficiency in Tsg101 causes an increase in the expression of LC3 and an enrichment of this protein within distended lysosomes, which all are indicative of the induction of macroautophagy.

### Tsg101-deficient cells initiate autophagy to extend their survival before they undergo apoptosis

**Figure 5:**
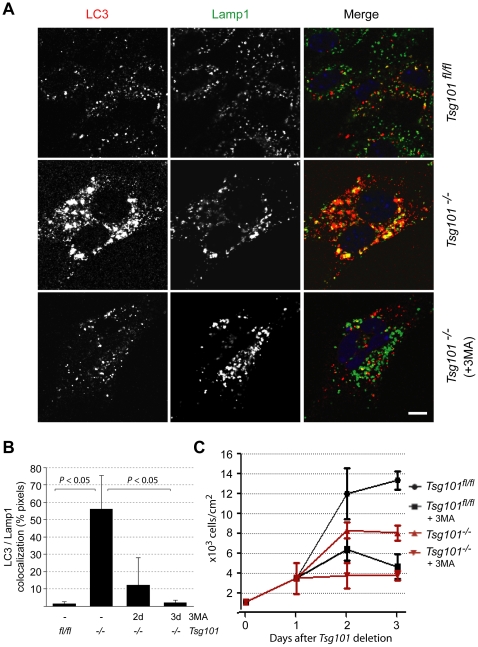
Tsg101-deficient cells initiate autophagy to prolong their survival prior to cell death. **A**. Confocal images of wildtype cells and Tsg101 conditional knockout cells that were grown in the presence or absence of 3-Methyladenine (3MA) to inhibit the fusion and maturation of LC3 containing autophagosomes with lysosomes. Tsg101-deficient cells were maintained in the presence of the inhibitor following one day after deletion of *Tsg101* using an adenovirus-based delivery of Cre recombinase; bar represents 10 µm. **B**. Quantitative analysis of the colocalization of LC3 and Lamp1 following 2 and 3 days of treatment with 3MA (*P* value, *t* test). **C**. Counts of Tsg101-deficient cells and their controls following treatment with 1.5 mM 3MA. The inhibitor was administered 24 hrs after infection with AdCre and deletion of *Tsg101*.

The inclusion of LC3 as well as the Cathepsin D protease into the greatly enlarged lysosomes suggested that Tsg101 knockout cells contain functional autophagolysosomes that are able to sequester organelles and degrade their content. The formation of autophagosomes that subsequently fuse with endosomes and lysosomes to mature into autophagolysosomes is initiated by the class III PI3 kinase Vps34. Treatment of nutrient-deprived cells with a PI3 kinase inhibitor, 3-methyladenine (3MA), has been demonstrated to attenuate autophagy which subsequently leads to accelerated cell death [30]. To assess the importance of autophagy as a survival mechanism for stressed Tsg101-deficient cells, we treated these cells and their wildtype controls with 3MA to block the maturation of functional autophagolysosomes. The immunofluorescent staining of LC3 and Lamp1 in treated Tsg101-deficient cells compared to their untreated knockout controls showed that the PI3 kinase inhibitor led to a decrease in the expression and dissociation of LC3 from the lysosome (Fig. 5A, 5B). Despite the lack of co-localization of the two markers, some of the Lamp1-positive structures remained enlarged. Next, we examined whether blocking the formation of functional autophagolysosomes was sufficient to accelerate the death of Tsg101 knockout cells that are destined to succumb from the loss of this gene. Although treatment of wildtype fibroblasts with 1.5 mM 3MA showed some degree of toxicity, Tsg101-deficient cells were much more sensitive to the PI3 kinase inhibitor (Fig. 5B). The observation that the treatment of Tsg101-deficient cells with 3MA causes accelerated growth arrest and cell death may suggest that stressed Tsg101 knockout cells utilize autophagy as a mechanism to prolong their survival prior to their ultimate demise.

**Figure 6:**
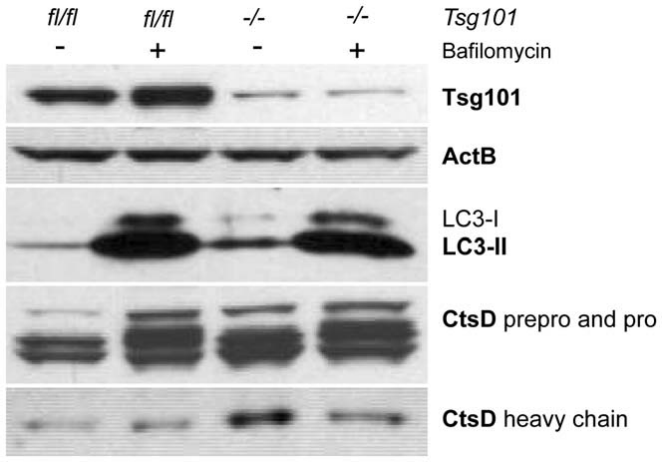
Tsg101 knockout cells exhibit increased expression of LC3 and activated Cathepsin D. Western blot analysis of LC3 as well as active Cathepsin D (CtsD) and its unprocessed precursors in Tsg101 knockout cells and their controls. Both types of cells were treated with bafilomycin A1 to assess the functionality autophagolysosomes. Note that treatment of Tsg101-deficient cells with this inhibitor, which prevents the acidification of lysosomes, reduces the level of processed Cathepsin D.

In a complementary study, we treated cells with bafilomycin A1 to further assess the correct functionality autophagolysosomes. Bafilomycin A1 is a vacuolar type H^+^-ATPase inhibitor that suppresses macroautophagy by preventing acidification of lysosomes, which, in turn, causes an increase in LC3 and reduces the cleavage and activation of Cathepsin D. As shown in Fig. 6, loss of Tsg101 resulted in an increase in the steady state levels of LC3 (both LC3-II and its precursor LC3-I) as well as Cathepsin D (CtsD), in particular the activated form (CtsD, heavy chain). As expected, treatment of these knockout cells and wildtype controls with bafilomycin A1 increases the expression of LC3 and mainly the unprocessed precursors of Cathepsin D (CtsD, prepro and pro). The reduction in the activated form of Cathepsin D in Tsg101-deficient cells that were treated with bafilomycin A1 compared to knockout cells without the inhibitor indicates that the autophagolysosomes in the Tsg101-deficient cells are functional.

### Expression of exogenous Tsg101 in knockout cells reverses the autophagic process

**Figure 7:**
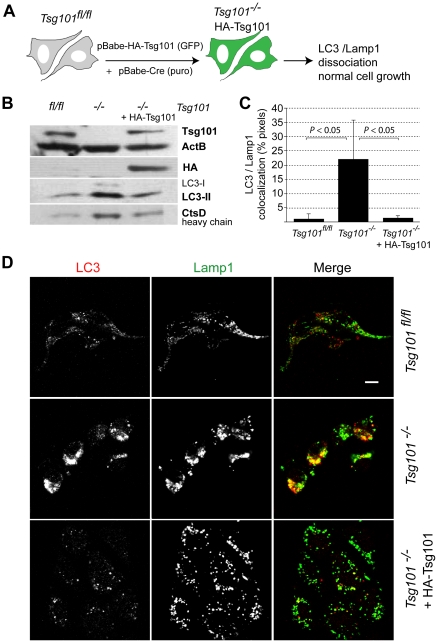
Permanent rescue of Tsg101 knockout cells from the stress-induced induction of autophagy through expression of exogenous, HA-tagged Tsg101 from a retroviral vector. **A**. Experimental design. **B**. Western blot analysis to verify the reduction in the levels of LC3 and Cathepsin D (CtsD) upon re-expression of Tsg101. **C**., **D**. Analysis of confocal images of Tsg101 conditional knockout cells with and without exogenous HA-tagged Tsg101 and their wildtype controls that were co-stained with antibodies against endogenous LC3 and Lamp1. Panel C illustrates the quantitative analysis of the colocalization of LC3 and Lamp1 (*P* value, *t* test). Bar in panel D represents 10 µm.

We have shown previously that re-expression of an HA-tagged Tsg101 protein in conditional knockout cells that lack both endogenous *Tsg101* alleles lifts the cell cycle block and promotes their survival [12]. We repeated this rescue experiment as illustrated in Fig. 7A to assess whether the induction of autophagy can be averted through expression of exogenous Tsg101 in the complete knockout background. The results of this study demonstrated that reinstating the functionality of Tsg101 lowers the level of the autophagy-associated protein LC3 and reduces the expression of active Cathepsin D (Fig. 7B). Finally, we used immunofluorescence staining of LC3 and Lamp1 to verify that restoring the expression of exogenous Tsg101 causes a dissociation of any remaining LC3 from the lysosome. The confocal analysis clearly showed that the intense co-localization of both markers that we repeatedly observed in Tsg101 knockout cells was absent when the expression of Tsg101 was reinstated (Fig. 7C, 7D). This suggests that the induction of autophagy is a direct and reversible cellular stress response that is caused by the loss of Tsg101.

## Discussion

Although cause and phenotypic consequences of a complete ablation of Tsg101 are difficult to discriminate due to the complexity of the intracellular changes, our study provides evidence that deficiency in Tsg101 leads to a gradual decrease in the steady-state levels of the EGFR and ErbB2 within a period of 4 days when a significant number of Tsg101 knockout cells remained viable. At no given time point did we observe an increase in the expression of the EGFR that would indicate that loss of Tsg101 enhances the recycling of the receptor back to the cell membrane or promotes a retention of the EGFR within the endosomal compartment as previously suggested [10], [23], [24]. More importantly, since ErbB2 does not normally traffic to lysosomes and is thought to primarily recycle, our findings that Tsg101 deficiency results in lower expression levels of this receptor raises the possibility that Tsg101 may regulate sorting processes distinct from the known effects of Tsg101 on promoting ubiquitinated cargo sorting into multivesicular bodies.

Similar to a knockout of Tsg101, the genomic deletion of the genes encoding the EGFR and ErbB2 causes embryonic lethality in mice [31], [32]. It can therefore be assumed that a substantial decline in the steady state levels of both receptor tyrosine kinases in response to Tsg101 deficiency acts as a cellular stressor that is comparable to growth factor deprivation. In our previous studies and in this report, we provide several lines of evidence that Tsg101 deficiency leads to an extensive cellular stress response that is characterized by 1) a p53-mediated cell cycle arrest, 2) the induction of stress kinases in the absence of growth factors, 3) an excessive remodeling of the actin filaments, as well as, 4) an enlargement of lysosomes and induction of autophagy. These complex changes in various molecular pathways need to be recognized as they can have a significant impact on defining particular functions for Tsg101 in processes such as protein sorting, trafficking, and signal transduction. For example, the intracellular targeting of activated Src is dependent on the actin cytoskeleton [33]. Since deficiency in Tsg101 causes a widespread redistribution of actin filaments, this might, in part, explain why Tsg101 is important for targeting Src to focal adhesions [34].

We observed that Tsg101 knockout cells contain greatly distended Lamp1-positive lysosomes. Although we did not examine the specific kinetics of the routing of biosynthetic cargo and nascent lytic hydrolases, it is evident from the results of this study that Tsg101 is not essential for the delivery of cargo such as Cathepsin D to the lysosome as reported previously [10]. In addition, the lysosomal compartment in Tsg101 knockout cells appears to be functional based on the correct cleavage and activation of Cathepsin D. The normal processing of this protease in the Tsg101 knockout cells could be inhibited through administration of bafilomyin A1, which is known to increase the pH in lysosomes and endosomes [35]. It has been proposed recently that the various ESCRT complexes including Tsg101 are required for the autophagic degradation of certain protein aggregates that are associated with neurodegenerative diseases [25]. While depletion of Tsg101 with siRNAs did not lead to an increase in *Map1lc3* mRNA expression, the level of the LC3-II protein was elevated in HeLa cells where Tsg101 was knocked down. Expression of an LC3-GFP fusion protein also led to the formation of large aggregates with p62, and it was suggested that this was the result of an inhibition of the degradation of both proteins due to impaired autophagolysosome formation. It cannot be excluded that these observed abnormalities might specific for HeLa cells or the type of functional ablation of the Tsg101 protein in these and other cells. The conditional knockout of the *Tsg101* gene on the genomic level in normal fibroblast did not block the delivery and Cathepsin D to the lysosome and its processing at this cell organelle, and, more importantly, the extensive co-localization between the lysosomal protein Lamp1 with endogenous LC3 (see Figs. 3C, 5A, and 7) suggests that lack of Tsg101 did not prevent the fusion between autophagosomes and lysosomes. This assumption is supported by the fact that Tsg101 conditional knockout cells increase the expression of LC3 on the transcriptional level in response to cellular stress, and they use autophagy to extend their survival prior to cell death.

As our previous studies and this report show, the G_1_/S cell cycle arrest, the induction of autophagy, and survival of cells that lack both copies of the endogenous *Tsg101* gene can be completely and permanently rescued through expression of exogenous wildtype Tsg101 from a retrovial vector. At this point, it is still unclear, which of the proposed multifaceted functions of Tsg101 are actually essential for the survival and normal homeostasis of a mammalian cell. Also, the biological relevance of the interaction between Tsg101 and other proteins including members of the ESCRT-1 complex and suggested ubiquitinated cargo proteins has not been examined *in vivo*. The more in-depth phenotypic characterization of Tsg101 conditional knockout cells on the intracellular level is a first step in this direction. Since the levels of Tsg101 are precisely controlled within a narrow range in normal cells [12], [14], the rescue experiment described in this study leads to the generation of stable cell lines that only express exogenous Tsg101 at physiologically relevant levels. This methodology is now being employed to assess the importance of particular domains of the Tsg101 protein through expression of mutant forms of this protein in cells that lack both endogenous *Tsg101* alleles.

## Materials and Methods

### Cell cultures and Cre-mediated deletion of Tsg101

The generation and propagation of immortalized mouse embryonic fibroblast (MEFs) from 13.5 or 14.5-day-old *Tsg101^fl/fl^* embryos was described previously [12], [14]. Forty-eight hrs after infection of these cells with a pBabe-Cre-puro retroviral vector or pBabe-puro control vector, cells were selected in complete medium containing 7 µg/ml puromycin (Sigma) as described [12], [13]. For the adenoviral-based delivery of Cre recombinase, cells were infected with the AdCre vector at an MOI of 5 in regular DMEM for 90 min with frequent agitation. Fresh media was added and the cells were incubated overnight. The AdCre containing media was replaced the next morning.

### Western blot analysis

A detailed description of the preparation of whole-cell extracts of clarified cell lysates and experimental procedures for western blot analyses was described earlier [12], [14]. The following antibodies were used in this study: α-Tsg101 (C-2) (1∶1000 dilution), α-ActB (I-19)(1∶1000 dilution) and α-EGFR p-Tyr1173 (sc-12351) from Santa Cruz Biotechnology; α-EGFR (1∶200 dilution) from AbCam; α-ErbB2 (Her2/Neu) (OP-15) (1∶1000 dilution) from Calbiochem; α-phospho-ERK (1∶5000 dilution) from Cell Signaling Technology, α-pan-ERK (1∶2000 dilution) from BD Bioscience; and α-Map1LC3 (4E12) (1∶2000 dilution) from MBL International. Horseradish peroxidase-conjugated secondary antibodies were purchased from Santa Cruz Biotechnology and used at a dilution of 1∶1000. The Cathepsin D antibody was a generous gift from Dr. Kurt von Figura, President of the Georg-August-Universität Göttingen, Germany.

### RNA extraction, semi-quantitative and quantitative RT-PCR

Total RNA was extracted from cells using standard guanidinium thiocyanate-phenol-chloroform extraction. A Superscript II kit from Invitrogen with Oligo(dT) primers was utilized to perform the fist strand synthesis. PCR amplification of the mouse *Epidermal Growth Factor Receptor* (*EGFR*) transcript was performed using the following primer pair: 5′-AAT GGG AGC TGC CGT GTC AAA GA-3′ and 5′-TAA ACC CAC TAC TGA GAC AGG TA-3′. A semi-quantitative analysis of the PCR amplicons on agarose gels were performed after 24, 27, and 30 cycles. The expression of *Hypoxanthine Phosphoribosyltranferase* (*Hprt*) severed as loading control. Quantitative detection of *Map1lc3* was performed using the TaqMan Mastermix (Applied Biosystems, Foster City, CA) with addition of *Map1lc3*- and *Gapdh*-specific primers and probe sets (Mm007828698_sH and 4352339E). The quantitative PCRs (qPCRs) were carried out in triplicate in a CFX96 real-time PCR detection system (Bio-Rad, Hercules, CA). The expression values obtained were normalized against *Gapdh* standard curve generated from the same samples.

### Transfection of plasmid expression vectors

The mammalian expression vector for the EGFP-LC3 fusion protein [36] was obtained from Addgene (plasmid 11546). Cells were transiently transfected 48–72 hrs prior to confocal analysis using either FuGENE (Roche Diagnostics) or Lipofectamine (Invitrogen) reagents. The pcDNA3-hEGFR plasmid was a kind gift from William Muller (McGill University). For stable expression of the hEGFR, transfected cells were selected with 200 µg/ml hygromycin B.

### Immunofluorescent staining and confocal microscopy

Tsg101-deficient and control cells were grown on poly-L-lysine coated glass coverslips. Cells were fixed with a solution of 4% (v/v) paraformaldehyde in PBS for 10 min. Next, cells were blocked in staining solution (0.2% (w/v) saponin, 10% (w/v) bovine serum albumin/PBS) for 30 min before incubation with appropriate primary antibody for 45–60 min at room temperature. Primary antibodies used in these studies include α-Cathepsin D (1∶500), and α-Lamp1 (1∶800) antibody that was purchased from Santa Cruz Biotechnology. The Cathepsin D antibody was a generous gift from Dr. Kurt von Figura, President of the Georg-August-Universität Göttingen, Germany. The α-Map1lc3 (1∶500 dilution) antiserum was obtained from MBL International, and the Alexa Fluor 594-labeled phalloidin was purchased from Invitrogen. Coverslips were washed four times in 1× PBS before being incubated with an appropriate secondary antibody for 30 min. Corresponding secondary antibodies conjugated to Alexa Fluor dyes 405, 430, 488, 568, or 594 were purchased from Invitogen. Labeled anti-mouse and anti-rat antibodies were used at a 1∶800 dilution, while anti-rabbit antibodies were used at a 1∶1500 dilution. Coverslips were mounted on slides using about 25–50 µl of Vectashield mounting medium with 4′,6-diamidino-2-phenylindole (DAPI) (Vector) and sealed with clear nail polish. Cells were visualized with a LSM 5 Pascal confocal microscope (Carl Zeiss, Thornwood, NY) using a 63×1.4 numerical aperture objective and filters appropriate for the various fluorochromes used.

### Inhibition of autophagy using 3-Methyladenine and bafilomycin A1

Following adenoviral-mediated expression of Cre recombinase, Tsg101-deficient cells and their controls were maintained for 1, 2, and 3 days in the presence of 1.5 mM 3-Methyladenine (3MA). For confocal microscopic analysis, cells were fixed on the third day in 4% paraformaldehyde and stained with anti-Lamp1 and anti-LC3 antibodies. To assess the survival rate during a 3-day time course in the presence and absence of 3MA, Tsg101-deficient cells and their controls were seeded into 6-well plates at a density of 33,000 cells per well. Cells were harvested, stained with Trypan blue (Invitrogen), and viable cells were counted on the three consecutive days following administration of 3MA. To modulate the autophagic process using bafilomycin A1, cells were infected with AdCre and maintained in fresh growth media overnight. The growth media was replaced the next day, and 10 nM of bafilomycin A1 was added. Cells were harvested for western blot analysis after an overnight incubation with the drug.
